# Impact of body mass index on perioperative mortality of acute stanford type A aortic dissection: a systematic review and meta-analysis

**DOI:** 10.1186/s12872-023-03517-z

**Published:** 2023-10-31

**Authors:** Wenyu Song, Jiani Liu, Guowei Tu, Lulu Pan, Yixiang Hong, Lieyang Qin, Lai Wei, Jinmiao Chen

**Affiliations:** 1 Department of Cardiovascular Surgery, Zhongshan Hospital, Fudan University, Shanghai, China; 2 School of Public Health, Prince of Wales Hospital, The Chinese University of Hong Kong, Shatin, New Territories Hong Kong, China; 3 Cardiac Intensive Care Center, Zhongshan Hospital, Fudan University, Shanghai, China; 4 Department of Biostatistics, School of Public Health, Nanjing Medical University, Nanjing, China; 5 Department of Biostatistics, Emory University, Atlanta, GA USA

**Keywords:** Acute Stanford type A aortic dissection, Body mass index, Perioperative mortality, Systematic review, Meta-analysis

## Abstract

**Background:**

Obesity may increase perioperative mortality of acute Stanford type A aortic dissection (ATAAD). However, the available evidence was limited. This study aimed to systematically review published literatures about body mass index (BMI) and perioperative mortality of ATAAD.

**Methods:**

Electronic literature search was conducted in PubMed, Medline, Embase and Cochrane Library databases. All observational studies that investigated BMI and perioperative mortality of ATAAD were included. Pooled odds ratio (OR) and 95% confidence interval (CI) were calculated using a random-effects model. Meta-regression analysis was performed to assess the effects of different clinical variables on BMI and perioperative mortality of ATAAD. Sensitivity analysis was performed to determine the sources of heterogeneity. Egger’s linear regression method and funnel plot were used to determine the publication bias.

**Results:**

A total of 12 studies with 5,522 patients were eligible and included in this meta-analysis. Pooled analysis showed that perioperative mortality of ATAAD increased by 22% for each 1 kg/m^2^ increase in BMI (OR = 1.22, 95% CI: 1.10–1.35). Univariable meta-regression analysis indicated that age and female gender significantly modified the association between BMI and perioperative mortality of ATAAD in a positive manner (meta-regression on age: coefficient = 0.04, *P* = 0.04; meta-regression on female gender: coefficient = 0.02, *P* = 0.03). Neither significant heterogeneity nor publication bias were found among included studies.

**Conclusions:**

BMI is closely associated with perioperative mortality of ATAAD. Optimal perioperative management needs to be further explored and individualized for obese patient with ATAAD, especially in elderly and female populations.

**Trial registration:**

PROSPERO (CRD42022358619).

**Graphical Abstract:**

BMI and perioperative mortality of ATAAD.

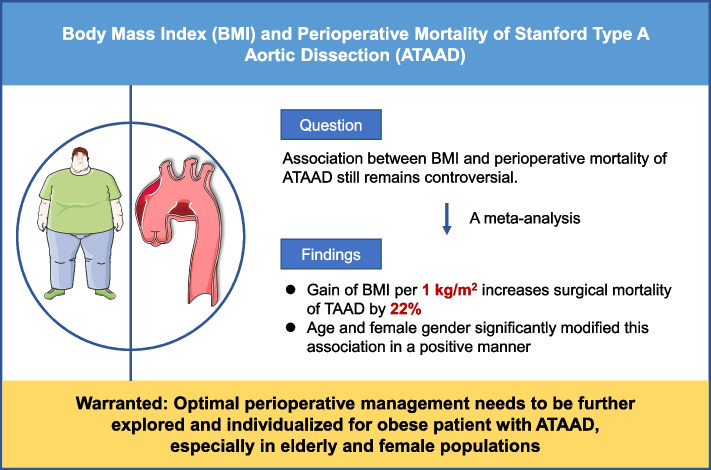

**Supplementary Information:**

The online version contains supplementary material available at 10.1186/s12872-023-03517-z.

## Introduction

Acute Stanford type A aortic dissection (ATAAD) is a devastating cardiovascular emergency that is triggered by internal and external factors and usually requires urgent surgical repair [1]. Despite the technical development, perioperative mortality of ATAAD remained as high as 9%–25% [2–5]. Therefore, identification of potential risk factors could improve personalized management of ATAAD among specific individuals.

Obesity is a critical issue of public health and is associated with the poor prognosis of many cardiovascular diseases [6]. Some studies reported that obesity raised the risk of perioperative mortality of ATAAD [7, 8]. However, other studies failed to demonstrate a significant association between body mass index (BMI) and perioperative mortality of ATAAD [9, 10]. Relatively low sample size and single-center design of most previous studies may contribute to controversial conclusions. Currently, solid evidence for the association between BMI and perioperative mortality of ATAAD is still lacking and evidence-based guidelines for management of obese ATAAD patients are also absent [11].

Herein, we systematically review published literature and performed this meta-analysis to explore the association between BMI and perioperative mortality of ATAAD. In addition, we assessed the effect of different clinical variables on this association.

## Methods and materials

### Search strategy

This meta-analysis was designed and conducted in the guidance of the Preferred Reporting Items for Systematic Reviews and Meta-Analyses (PRISMA) checklist [12]. In addition, we also followed the Meta-analysis of Observational Studies in Epidemiology (MOOSE) guidelines because the included studies were observational in design [13]. A systematic search of published articles was conducted in PubMed, Medline, Embase and Cochrane Library databases until December 2022. Combination of following terms were used: (1) “Type A aortic dissection” or “Stanford type A aortic dissection” or “ATAAD”; and (2) “BMI” or “Body Mass Index” or “Obesity” or “Body Weight”; and (3) “Mortality” or “Mortalities” or “Fatality Rate” or “Death Rate” or “Fatality Rates” or “Death Rates”. References from selected literatures were also manually scrutinized for potentially relevant citations using the snowball methodology [14]. The references were imported to EndNote online and Excel to remove duplicated records. Eligibility assessment of the first screening was performed based on titles and abstracts by three independent investigators (W.S., J.L. and L.P.). The second screening was conducted with the inclusion and exclusion criteria on the full text by all three investigators (W.S., J.L. and L.P.). In case of uncertainty, an agreement was negotiated and where necessary, a fourth researcher was consulted (J.C.).

### Inclusion and exclusion criteria

Studies meeting the following criteria were included in this meta-analysis: (1) Observational studies; (2) Patients were diagnosed as ATAAD regardless of age and underwent surgical repair; (3) Concerning the effect of BMI on perioperative mortality of ATAAD; (4) Publication in English. On the contrary, studies meeting the following criteria were excluded: (1) Duplicated records; (2) Failure to focus on BMI and perioperative mortality of ATAAD; (3) Concerning the effect of BMI on other outcomes instead of perioperative mortality; (4) Effect values of BMI on perioperative mortality of aortic dissection were not available. (5) Reviews, editorial comments, case reports, conference abstracts, and expert opinions; (6) Full text was not available.

### Data extraction

The following information was collected for each study enrolled in this meta-analysis: (1) First author’s name; (2) Region; (3) Study period; (4) Study design; (5) Sample size; (6) Female proportion; (7) Mean age; (8) Smoker proportion; (9) Hypertension proportion; (10) Diabetes proportion; (11) Obesity measure; (12) Effect values with 95% confidence intervals (CIs).

### Definitions and endpoints

All cases of ATAAD were clearly diagnosed using computed tomography angiography in 12 enrolled studies [1]. Perioperative mortality was defined as in-hospital mortality or 30-day mortality of ATAAD patients after surgical repair.

### Quality assessment

The quality assessment was performed by three independent investigators (W.S., J.L. and L.P.). A fourth investigator was consulted in cases of uncertainty (J.C.). Quality assessment was conducted with the Newcastle–Ottawa Quality Assessment Scale (NOS), a validated scale for non-randomized observational studies [15]. Case–control and cohort study were assessed according to three aspects: (1) selection (0–4 points); (2) comparability (0–2 points); (3) exposure/outcome (0–3 points). Higher total scores represented higher quality. The acceptable score was at least 6.

### Statistical analysis

Odds ratio (OR) was calculated according to its definition if effect value was not given directly. In order to set an unified standard of effect values, hazard ratio (HR) was directly regarded as OR according to a previous meta-analysis [16]. Adjusted OR and 95% CI were used to evaluate the association between BMI and perioperative mortality of ATAAD. To investigate the potential sources of result variations, we conducted univariable meta-regression analysis to explore whether the research results were influenced by different clinical variables and participant characteristics. Heterogeneity among studies was evaluated through Q statistic. Given that heterogeneity was nonnegligible between only 12 studies included in this meta-analysis, a random-effects model was applied to calculate the combined effect value compared with fixed effects models according to our previous study [17]. Further, a one-by-one elimination method was used to perform sensitivity analysis. Egger’s linear regression method was used to check publication bias. Statistical analyses were conducted through STATA 16.0 (Stata Corp, Texas, USA). Statistical significance was set at *P* < 0.05. All tests were two-sided.

## Results

### Literature selection

**Fig. 1:**
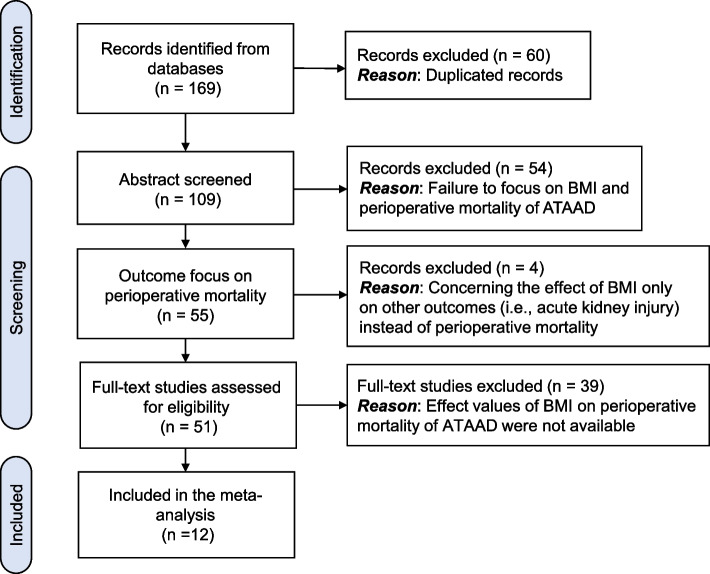
The flow chart of the study procedure

The literature search identified 169 records from databases concerning effects of BMI (or obesity) on perioperative mortality of ATAAD. After further exclusions, 12 literatures met our selection criteria and were included for this meta-analysis. The flow chart of the literature search was shown in Fig. 1.

### Study characteristics and quality assessment

Basic characteristics of the studies included in this meta-analysis were shown in Table 1. A total of 12 observational studies included 4 case–control and 8 cohort studies. Of these studies, 8 were performed in China, 2 in Japan, 1 in Germany and 1 in Italy. Sample size ranged from 72 to 1,059. Percent of female patients ranged from 12.5% to 48.0%. Mean age of patients ranged from 46.0 to 65.7 years. Clinical characteristics of these studies were shown in Table 2. The percent of smokers, hypertension and diabetes mellitus ranged from 31.3% to 54.2%, 46.0% to 88.2%, and 2.2% to 54.0%, respectively. Five studies used mean BMI, while six studies divided the BMI into two categories by the cut-off value of 25.0 or 30.0 kg/m^2^. One study classified patients into four groups: normal weight (18.0 ≤ BMI < 25.0 kg/m^2^), overweight (25.0 ≤ BMI < 30.0 kg/m^2^), obese (30.0 ≤ BMI < 35.0 kg/m^2^) and morbidly obese (BMI ≥ 35.0 kg/m^2^). NOS scores of all 12 studies ranged from 6 to 8 (Tables 3 and 4).

**Table 1:** Basic characteristics of the studies included in the meta-analysis

Study	Region	Study period	Study design	Sample size	Female n (%)	Mean age (years)
Huo Y. et al., 2021 [18]	China	2016–2018	Case control, Retrospective	149	42 (28.2)	51.4
Kreibich, M. et al.,2018 [9]	Germany	2003–2017	Cohort, Retrospective	667	220 (33.0)	59.4
Zhang, Y. H. et al.,2021 [19]	China	2017–2020	Case control, Retrospective	224	52 (23.2)	52.8
Shimizu, T. et al.,2020 [8]	Japan	1990–2018	Cohort, Retrospective	1,059	504 (47.6)	65.7
Wu Y. H. et al.,2018 [20]	China	2010–2016	Case control, Retrospective	72	9 (12.5)	47.6
Kawahito, K. et al., 2017 [7]	Japan	1990–2016	Cohort, Retrospective	1,026	492 (48.0)	64.3
Wang M. Z. et al., 2021 [21]	China	2018–2019	Cohort, Retrospective	931	172 (18.5)	48.9
Lio A et al., 2019 [22]	Italy	2006–2013	Cohort, Retrospective	201	54 (26.9)	62.4
Pan X. G. et al., 2022 [23]	China	2014–2016	Cohort, Retrospective	289	74 (25.6)	48.6
Liu Y. X. et al., 2021 [10]	China	2017–2019	Cohort, Retrospective	268	54 (20.1)	46.0
Luo Z. R. et al., 2021 [24]	China	2017–2019	Cohort, Retrospective	258	70 (27.1)	53.9
Lin Y. J. et al., 2022 [25]	China	2018–2020	Case control, Retrospective	378	73 (19.3)	47.9

**Table 2:** Clinical characteristics of the subjects in the included studies

Study	Smoker n (%)	Hypertension n (%)	Diabetes n (%)	Obesity measure (kg/m2)	Effect values (95% CI)
Huo Y. et al., 2021 [18]	69 (46.3)	110 (73.8)	4 (2.7)	BMI > 25.0	OR = 7.52 (1.37–41.36)
Kreibich, M. et al.,2018 [9]	NA	588 (88.2)	71(10.6)	Normal weight (18.0 ≤ BMI < 25.0)	
				Overweight (25.0 ≤ BMI < 30.0)	OR = 0.91 (0.43–1.91)
				Obese (30.0 ≤ BMI < 35.0)	OR = 1.27 (0.57–2.83)
				Morbidly obese (BMI ≥ 35.0)	OR = 1.21 (0.48–3.08)
Zhang, Y. H. et al.,2021 [19]	108 (48.2)	156 (69.6)	5 (2.2)	Mean BMI	OR = 1.15 (1.03–1.29)
Shimizu, T. et al.,2020 [8]	339 (32.0)	761 (71.9)	70 (6.6)	Obese (BMI ≥ 30.0)	OR = 3.00 (1.40–6.20)
Wu Y. H. et al.,2018 [20]	NA	53 (73.6)	NA	Mean BMI	OR = 1.32 (1.03–1.70)
Kawahito, K. et al., 2017 [7]	329 (32.1)	NA	66 (6.4)	Obese (BMI ≥ 30.0)	OR = 3.16 (1.48–6.74)
Wang M. Z. et al., 2021 [21]	NA	687 (73.8)	30 (3.2)	Mean BMI	HR = 1.13 (1.03–1.25)
Lio A et al., 2019 [22]	63 (31.3)	175 (87.1)	9 (4.5)	Obese (BMI ≥ 30.0)	OR = 2.14 (1.22–3.78)
Pan X. G. et al., 2022 [23]	NA	133 (46.0)	156 (54.0)	Obese (BMI ≥ 30.0)	HR = 2.61 (1.30–5.22)
Liu Y. X. et al., 2021 [10]	NA	222 (82.8)	8 (3.0)	Obese (BMI ≥ 30.0)	OR = 0.78 (0.18–3.35)
Luo Z. R. et al., 2021 [24]	NA	216 (83.7)	43 (16.7)	Mean BMI	HR = 1.10 (0.96–1.12)
Lin Y. J. et al., 2022 [25]	205 (54.2)	327 (86.5)	49 (13.0)	Mean BMI	OR = 1.09 (1.04–1.15)

**Table 3:** Study quality of case–control studies

	Selection				Comparability	Exposure			
Study	Case definition	Representativeness of the cases	Selection of Controls	Definition of Controls		Ascertainment of exposure	Same method of ascertainment for cases and controls	Non-Response rate	Total score
Zhang, Y. H. et al.,2021 [19]	*	*	*	*	**	*	*	-	8
Wu Y. H. et al.,2018 [20]	*	*	*	-	*	*	*	-	6
Huo Y. et al., 2021 [18]	*	*	*	-	*	*	*	-	6
Lin Y. J. et al., 2022 [25]	*	*	*	*	**	*	*	-	8

Each one asterisk (*) represents one point in the quality assessment

**Table 4:** Study quality of cohort studies

	Selection				Comparability	Outcome			
Study	Representativeness of the exposed cohort	Selection of the nonexposed cohort	Ascertainment of exposure	Demonstration that outcome of interest was not present at start of study		Assessment of outcome	Was follow up long enough for outcomes to occur	Adequacy of follow up of cohorts	Total score
Wang M. Z. et al., 2021 [21]	*	*	*	*	**	*	*	-	8
Kreibich, M. et al.,2018 [9]	*	*	*	*	**	*	-	-	7
Shimizu, T. et al.,2020 [8]	*	*	*	*	*	*	-	-	6
Kawahito, K. et al., 2017 [7]	*	*	*	*	**	*	*	-	8
Lio A et al., 2019 [22]	*	*	*	*	*	*	*	-	7
Pan X. G. et al., 2022 [23]	*	*	*	*	**	*	*	-	8
Liu Y. X. et al., 2021 [10]	*	*	*	*	*	*	*	-	7
Luo Z. R. et al., 2021 [24]	*	*	*	*	**	*	*	-	8

Each one asterisk (*) represents one point in the quality assessment

### Association between BMI and Perioperative Mortality of ATAAD

The effect of BMI on perioperative mortality of ATAAD was extracted from 12 studies which included 5,522 patients. Single-center studies in China observed that mean BMI was associated with perioperative mortality of ATAAD, including 30-days mortality [21] (HR = 1.13, 95% CI: 1.03–1.25), in-hospital mortality [19, 20, 23, 25] (OR = 1.15, 95% CI: 1.03–1.29, OR = 1.32, 95% CI: 1.03–1.70, HR = 2.61, 95% CI: 1.30–5.22 and OR = 1.09, 95% CI: 1.04–1.15). Consistently, different perioperative mortality of ATAAD was found between different BMI categories. For example, a retrospective study in China observed significantly increased perioperative mortality in overweight patients [18] (BMI > 25.0 kg/m^2^, OR = 7.52, 95% CI: 1.37–41.36). Moreover, 2 independent retrospective cohort studies in Japan [7, 8] and 1 in Italy [22] identified obesity (BMI ≥ 30.0) as a risk factor of in-hospital mortality in ATAAD patients who underwent surgical repairs (OR = 3.00, 95% CI: 1.40–6.20, OR = 3.16, 95% CI: 1.48–6.74, and OR = 2.14, 95% CI: 1.22–3.78). However, a retrospective cohort study conducted in Germany [9] and 2 in China [10, 24] found no associations.

**Fig. 2:**
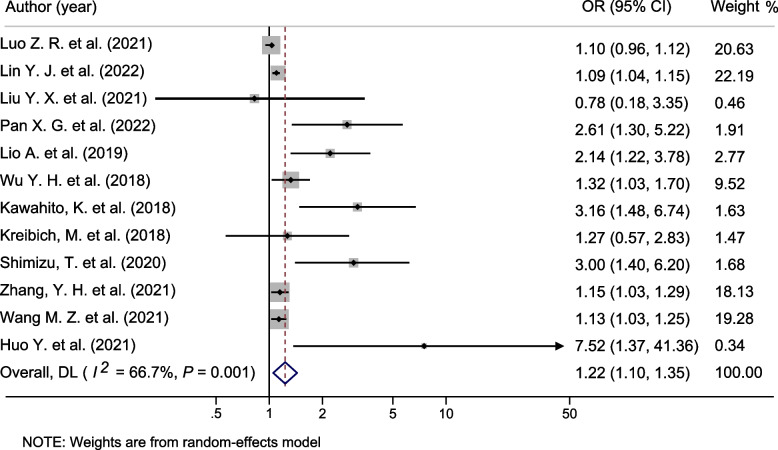
Effect of BMI on perioperative mortality of ATAAD

Through integration of these studies by a random-effects model, perioperative mortality of ATAAD increased by 22% for each 1 kg/m^2^ increase in BMI (Fig. 2, OR = 1.22, 95% CI: 1.10–1.35). Due to the low sample study of Wu Y. H. et al. [20], there may be potential publication bias. We added a forest plot excluding the research of Wu Y. H. et al., and the results still show the significant association between BMI and perioperative mortality of ATAAD (Figure S1, OR = 1.21, 95%CI: 1.09–1.35).

### Meta-regression

The meta-regression analysis was conducted to assess the impact of four continuous variables on the association between BMI and perioperative mortality of ATAAD, including age, gender, hypertension, and diabetes (Table 5). The results demonstrated that each 1-unit increase in age was associated with a 0.04-unit increase in the effect of BMI on the perioperative mortality on ATAAD (regression coefficient = 0.04, *P* = 0.04). Similarly, a 1-unit increase in the proportion of females was associated with a substantial 0.02-unit increase in the effect (regression coefficient = 0.02, *P* = 0.03). However, we found no statistically significant association between the proportion of hypertension patients (regression coefficient = -0.01, *P* = 0.27) or diabetes patients (regression coefficient = 0.01, *P* = 0.55) and the effect.

**Table 5:** Univariable meta-regression of four variables on the risk of perioperative mortality of ATAAD

Variables	Regression coefficient	95% CI	Standard Error	t	*P* >|t|
Mean age (years)	0.04	0.00–0.08	0.02	2.35	0.04
Female	0.02	0.00–0.05	0.01	2.51	0.03
Diabetes	0.01	-0.02–0.03	0.01	0.63	0.55
Hypertension	-0.01	-0.04–0.01	0.01	-1.20	0.27

### Sensitivity analysis

**Fig. 3:**
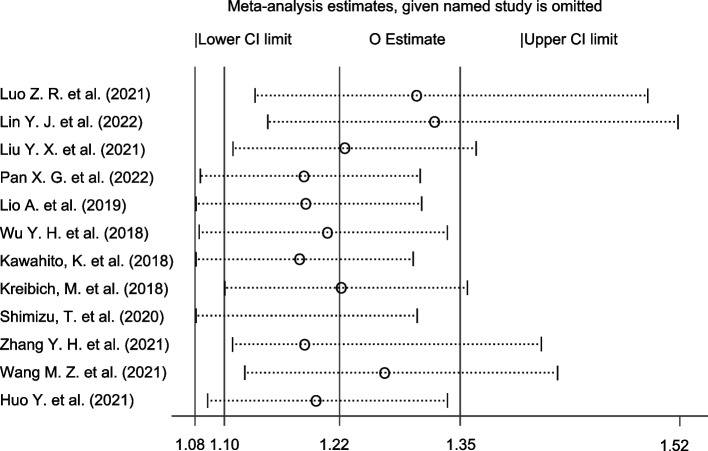
Sensitivity analysis of BMI on perioperative mortality of ATAAD

Sensitivity analysis was performed in Fig. 3. The pooled OR and 95% CI did not show evident differences after excluding each individual article one by one, which indicated that the studies included in our meta-analysis were credible.

### Publication bias

**Fig. 4:**
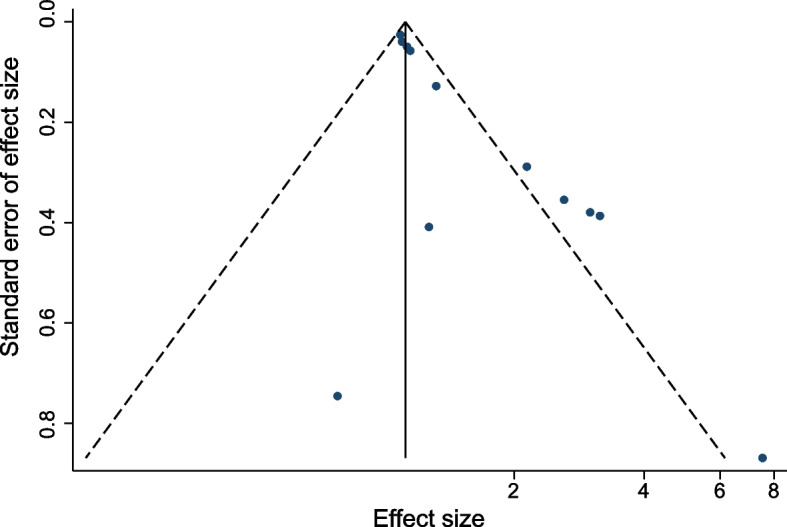
Funnel plot of the BMI on perioperative mortality of ATAAD

Publication bias was assessed with Egger’s test and funnel plot. Our statistical test showed no evidence of publication bias (Egger’s test *P* = 0.001). However, five points fall outside, suggesting the possibility of heterogeneity (Fig. 4). Too few studies were included which may lead to the biased result.

## Discussion

Over the decades, many risk factors have been identified in the morbidity and mortality of ATAAD [26–28]. A recent meta-analysis identified five risk factors for early death after surgery in patients with ATAAD, including age, gender, shock, malperfusion and cardiac tamponade [29]. However, there is still no high-quality evidence on the association between BMI and perioperative mortality of ATAAD. In this meta-analysis, we observed a positive association between BMI and perioperative mortality of ATAAD through integration of 12 independent studies (Graphic Abstract).

In clinical practice, surgical repair of ATAAD on obese patients is difficult and challenging. It usually required prolonged operation time, cardiopulmonary bypass time and myocardial ischemia time compared with non-obese individuals [22, 30–33]. These patients might suffer from multiple complications including cardiopulmonary dysfunction, multiple organ dysfunction syndrome and severe surgical site infection after surgical repair [22, 30, 34]. It was reported that obese ATAAD patients had a prolonged ICU stay (9 days) compared with non-obese patients (6 days) [8]. Meanwhile, the incidence of ECMO usage and renal replacement therapy increased from 2.4% and 7.1% in non-obese to 8.7% and 14.5% in obese ATAAD patients, respectively^8^. The difficulty of surgical repair and postsurgical management indicated that BMI might also be a major concern in perioperative mortality of ATAAD.

Respiratory dysfunction including preoperative [35] and postoperative hypoxemia [36] was a common complication of obese ATAAD patients undergoing surgical repair. The underlying mechanism might be multifactorial. Obese patients have lower functional residual capacity (FRC) secondary to cephalad diaphragmatic displacement. A low FRC increases the risk of both expiratory flow limitation and airway closure [37]. Biologically, obesity patients share systemic chronic inflammation in vivo compared to control individuals [38]. Under chronic persistent hypoxia, adipose tissue produces reactive oxygen species [39] and inflammatory factors [40, 41] into blood circulation including TNF-α, IL-1β and IL-6. It further leads to inflammatory reaction and oxidative stress which may contribute to increased lung injury [35, 42–44]. Resultantly, prolonged ventilation time and increased pulmonary morbidity were observed after cardiac surgery on obese patients [31–33]. In-depth understanding of cellular and molecular mechanisms could facilitate us to treat respiratory complication after surgical repair for obese ATAAD patients.

Other potential mechanisms may be used to explain the relation between BMI and perioperative mortality of ATAAD. Obesity is closely associated with various types of cardiovascular disorders such as hypertension, coronary heart disease and cerebrovascular disease. Hypertension incidence was 87.0% in obese ATAAD patients compared with 68.9% in non-obese individuals [8]. In addition, higher incidence of hemodynamic instability was found among obese ATAAD patients during perioperative periods [8]. These cardiovascular dysfunctions might increase the difficulty of perioperative management of ATAAD patients.

Given that obesity might increase perioperative mortality of ATAAD, obese patients with ATAAD should be specially focused and treated. Ventilation management is an important concern of perioperative management of obese ATAAD patients. Positive end-expiratory mandatory ventilation was recommended for obese patients at different phases of surgery including before intubation, on mechanical ventilation and after extubation [45]. Considering higher incidence of infection and hemodynamic instability among obese ATAAD patients, rational administration of antibiotics and vasoactive agents also needs to be focused during the perioperative management [22, 30]. In addition, high incidence of obstructive sleep apnea syndrome was found among obese ATAAD patients [9, 46], which indicated that personalized airway assessment, choice of anesthesia and analgesia technique and extubating time are also important for perioperative management of these patients [47, 48].

Meta-regression analysis further indicated that the effect of BMI on perioperative mortality of ATAAD might be stronger in elderly and female populations. Elderly patients should be paid more attention in clinical management due to the decreased metabolic and immune function, degraded physiological function of organs, poor body reserve and compensation ability in these populations [49, 50]. The impact of gender on perioperative mortality of ATAAD still remains controversial. Several studies have reported worse mortality for women who undergo surgery for aortic dissection [51–54], while other studies drew the opposite conclusion [29]. Pooled analysis demonstrated that female gender was not associated with increased perioperative mortality of ATAAD [55]. However, few studies have focused on the impact of gender on the association between BMI and perioperative mortality of ATAAD. Our meta-regression analysis indicated that female obese ATAAD patients may have increased risk of perioperative mortality than male obese ATAAD patients, which called for specialized management of female obese ATAAD patients.

The present study has some limitations. First, only 12 literatures were included in the study. Different study designs, classification criteria of BMI and outcome indicators may contribute to heterogeneity in this meta-analysis. Some original research literature might not reach enough high quality due to limited sample volume. Second, different countries may have different definitions on obesity. Thus, we mainly analyzed the relationship between perioperative mortality and the gain of BMI. Here, BMI was regarded as a continuous variable. In addition, different methods of surgical repairs of ATAAD were performed in these studies, such as Sun’s operation, David operation and Bentall operation, which could not be adjusted only through statistical manners.

## Conclusions

BMI is closely associated with perioperative mortality of ATAAD. Optimal perioperative management needs to be further explored and individualized for obese patient with ATAAD, especially in elderly and female populations.

### Supplementary Information

**Additional file 1.**

**Additional file 2:** **Figure S1.** Effect of BMI on perioperative mortality of ATAAD (exclude a low sample study).

## Data Availability

The datasets used and/or analyzed during the current study are available from the corresponding author on reasonable request.
