# Supplementary figures and images for: Impact of body mass index on perioperative mortality of acute stanford type A aortic dissection: a systematic review and meta-analysis
Source: BMC Cardiovasc Disord. 2023 Oct 31;23:531. doi: 10.1186/s12872-023-03517-z (PMC10617194; doi:10.1186/s12872-023-03517-z)

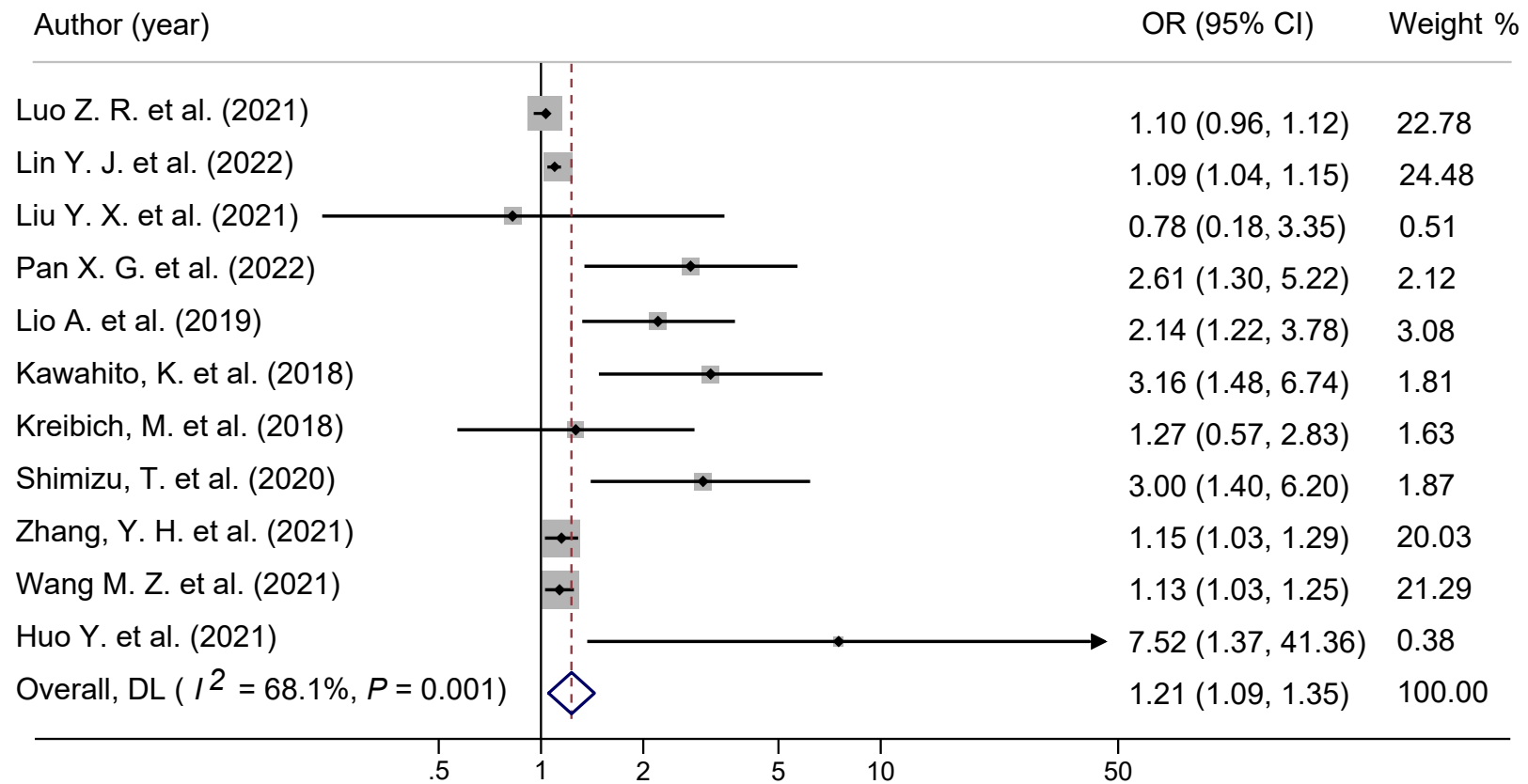

Supplement: Supplementary file 2 — Additional file 2: Figure S1. Effect of BMI on perioperative mortality of ATAAD (exclude a low sample study). [file 12872_2023_3517_MOESM2_ESM.pdf]
